# Disordered Eating Behaviours and Associated Factors Among People With Type 1 Diabetes: A Cross‐Sectional Study in Spain—D1ANAS Project

**DOI:** 10.1002/dmrr.70204

**Published:** 2026-07-15

**Authors:** Lorena Botella‐Juan, Inmaculada Aguilera‐Buenosvinos, María Antequera‐González, Laura Álvarez‐Álvarez, María Rubín‐García, María Romeo‐Santos, Ana Segura‐Grau, María D. Ballesteros‐Pomar, Antonio José Molina, Vicente Martín‐Sánchez

**Affiliations:** ^1^ Department of Biomedical Sciences, Area of Preventive Medicine and Public Health, Faculty of Health Sciences Universidad de León León Spain; ^2^ Consortium for Biomedical Research in Epidemiology & Public Health (CIBER Epidemiología y Salud Pública [CIBERESP]) Madrid Spain; ^3^ The Research Group in Gene‑Environment and Health Interactions (GIIGAS) Institute Biomedicine (IBIOMED), Universidad de León León Spain; ^4^ Faculty of Health Sciences International University of La Rioja Logroño Spain; ^5^ Servicio de Endocrinología y Nutrición Complejo Asistencial Universitario de León Gerencia de Salud de Castilla y León (SACYL) León Spain; ^6^ Hospital General Universitario Gregorio Marañón Madrid Spain; ^7^ Ultrasound Unit, Family and Community Medicine San Francisco de Asís Hospital Madrid Spain; ^8^ SEMERGEN (Spanish Society of Primary Care Physicians) Madrid Spain; ^9^ Instituto de Investigación Biosanitaria de León (IBIOLEON) León Spain

**Keywords:** diabetes mellitus, disordered eating behaviours, eating disorders, type 1 diabetes

## Abstract

**Background and Aims:**

People with type 1 diabetes mellitus (T1D) are at increased risk of disordered eating behaviours (DEBs), partly due to aspects of diabetes management. The aim of this study was to estimate the prevalence of DEBs in a group of adults with T1D in Spain and to examine their association with sociodemographic, clinical, and lifestyle‐related factors.

**Methods:**

A nationwide cross‐sectional study was conducted as part of the D1ANAS project, involving 451 people aged ≥ 16 years with T1D living in Spain. DEBs were assessed using the validated Spanish version of the Diabetes Eating Problems Survey‐Revised (DEPS‐R), with scores ≥ 20 indicating a positive screening. Sociodemographic, clinical, and lifestyle variables were collected using a self‐administered online questionnaire. Multivariate linear regression analyses were performed to identify factors independently associated with the total DEPS‐R score.

**Results:**

The mean DEPS‐R score was 16.8 (SD 12.1), and 31.7% of participants screened positive for DEBs, with higher prevalence in women than men (35.4% vs. 14.8%, *p* < 0.001) 36.6% of participants reported insulin restriction behaviours, with no significant differences between sexes. In the adjusted models, female sex (*β* = 3.23, 95% CI = 0.65−5.82), higher HbA1c percentage (*β* = 2.12, 95% CI = 1.32−2.93), and higher zBMI (*β* = 3.44, 95% CI = 2.44−4.44) were independently associated with higher DEPS‐R scores, as were poorer perceived health (*β* = 5.11, 95% CI = 2.25−7.96), poorer diet quality (*β* = 13.42, 95% CI = 7.23−19.61) and poor sleep perception (*β* = 3.43, 95% CI = 1.46−5.39). The model explained 29.6% of the variance in DEPS‐R scores.

**Conclusion:**

DEBs affect about one‐third of the participants with T1D and are associated with poorer metabolic control, higher BMI, and adverse lifestyle and psychosocial factors, highlighting the need for diabetes‐specific screening and multidisciplinary management.

## 1 Introduction

Type 1 diabetes mellitus (T1D) is a chronic autoimmune disease characterised by absolute insulin deficiency and the need for lifelong insulin replacement therapy. Optimal management of T1D requires sustained engagement in complex self‐care behaviours, including dietary planning, carbohydrate counting, insulin dose adjustment, and frequent monitoring of glycaemic control and body weight. While these strategies are essential to prevent acute and long‐term complications, they also require continuous attention to food intake, weight, and lifestyle behaviours, which may impose a substantial behavioural and psychosocial burden [1, 2].

There is robust evidence indicating that individuals with T1D are at increased risk of developing eating disorders (EDs) and disordered eating behaviours (DEBs) compared with the general population [2, 3]. Recent systematic reviews and meta‐analyses have consistently reported a higher prevalence of EDs, particularly bulimia nervosa and binge eating disorder, among people with T1D than among individuals without diabetes [3, 4]. In addition to classical ED psychopathology, diabetes‐specific behaviours such as intentional insulin restriction (RI) for weight control represent a distinctive and clinically relevant manifestation of DEBs in T1D [2, 5].

Several factors intrinsic to diabetes management have been proposed to contribute to the development and maintenance of these behaviours, including the constant focus on food intake, body weight, and glycaemic targets [1, 6]. Emerging evidence suggests that the continuous medical emphasis on dietary control and weight monitoring may inadvertently promote excessive preoccupation with eating and body shape in vulnerable individuals [7, 8]. This requirement for rigorous self‐monitoring may increase psychological vulnerability, as the cognitive and emotional demands of diabetes management overlap with core features of ED psychopathology [9, 10].

From an epidemiological perspective, prevalence estimates of DEBs in youths with T1D typically range from 20% to 35% [11, 12, 13]. In a nationwide Italian study [7] as well as in other Mediterranean cohorts, emotional and behavioural difficulties have been identified as key predictors of DEBs, suggesting that sociocultural and psychosocial factors may play an important role in this comorbidity [14, 15]. However, reported prevalence rates vary widely across studies depending on age, sex, assessment tools, and sociocultural context, highlighting the need for population‐specific data. Consistent sex differences have been reported, with a higher prevalence of DEBs among females with T1D, although males are increasingly recognised as an affected group [7, 11, 12, 13]. Importantly, evidence from large cohort studies indicates that these disturbances frequently persist into adulthood and remain significantly more prevalent among individuals with T1D than in non‐diabetic populations [16].

The identification of DEBs in individuals with T1D requires diabetes‐specific screening instruments. In this framework, the Eating Problems in Diabetes–Revised (DEPS‐R) questionnaire has demonstrated good psychometric properties in both adolescent and adult populations and is widely used in clinical and research settings [12, 13, 17].

Recent work by Merwin et al. further suggests that DEPS‐R scores capture heterogeneous behavioural profiles that are differentially associated with glycaemic outcomes in real‐world settings [6]. Beyond eating‐related behaviours, DEBs in adults with T1D are likely to coexist with lifestyle‐related factors, such as sleep disturbances and other health behaviours, which may further influence metabolic control and overall well‐being [3]. In line with this perspective, current international guidelines emphasise the importance of addressing psychosocial aspects as an integral component of comprehensive, patient‐centred diabetes care [18].

Despite their clinical relevance, population‐based data assessing the prevalence of DEBs and their associated factors in adults with T1D remain limited, particularly in Southern European populations such as Spain. Accordingly, the aim of the present study was to estimate the prevalence of disordered eating behaviours among a sample of Spanish people with T1D and to explore their associations with sociodemographic, clinical, and lifestyle‐related factors in a real‐world population.

## 2 Methods

### 2.1 Study Population

Participants were part of the D1ANAS project (*DIAbetes mellitus type 1, Nutrition and mental health, patient Associations*), a multicenter cross‐sectional study conducted in Spain in 2025 to assess the risk of DEBs among individuals with T1D. The study was designed to capture data from a broad, nationwide population of people with T1D in a real‐world setting. An important point is that the research team and collaborators on the D1ANAS project are made up of highly experienced researchers, clinical healthcare personnel, and patients with T1D and/or eating disorders (EDs). The inclusion of patients in the team provides a novel approach with potential benefits for the project and its scope. This initiative is based on the theoretical concept of PPI (Patient and Public Involvement) [19], based on the idea that research is carried out ‘with’ or ‘by’ members of the public rather than ‘about’ or ‘for’ them.

A convenience sampling strategy was employed. Recruitment was carried out primarily through dissemination of the study invitation via social media platforms and patient‐led networks, and was supported by a snowball approach, whereby participants were encouraged to share the survey with other individuals with T1D. This recruitment strategy allowed the inclusion of participants from different geographic regions across Spain.

Eligible participants were individuals aged 16 years or older with a diagnosis of T1D, Spanish‐speaking, living in Spain, and willing to provide informed consent. Individuals with other types of diabetes were excluded.

**FIGURE 1:**
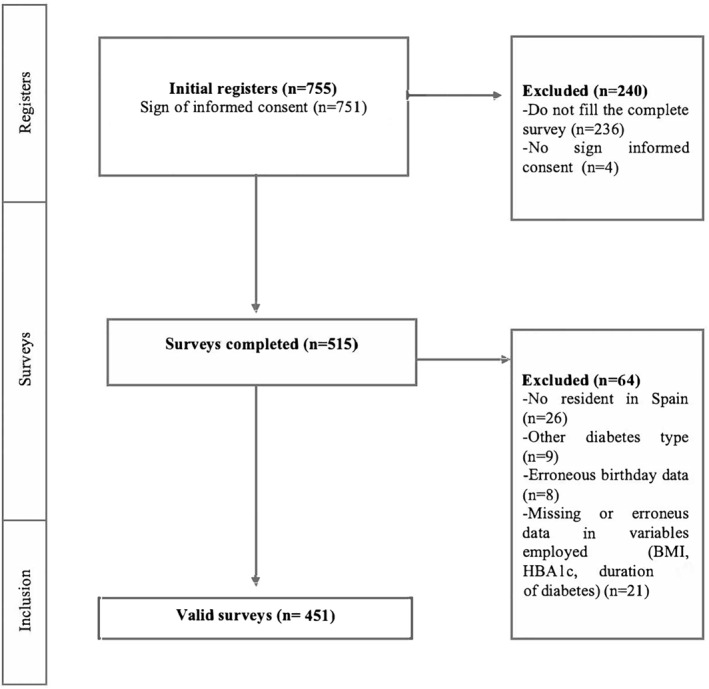
Flowchart diagram of the D1ANAS’ participants selection process.

The flow diagram of the participant selection process is detailed in Figure 1. Following the completion of the recruitment period, data were cleaned and screened for completeness and validity. A total of 755 questionnaires were initially registered, of which 751 participants provided informed consent. Of these, 515 questionnaires were fully completed. Subsequently, 43 questionnaires were excluded due to ineligibility criteria and another 21 questionnaires due to missing or implausible values in key variables. Following the data quality control procedures, a total of 451 valid questionnaires were retained and included in the final analytical dataset. Participants were spread across all of Spain's autonomous communities (with the exception of Ceuta and Melilla), with the highest numbers of participants coming from: Madrid (*n* = 79), Andalusia (*n* = 77), Catalonia (*n* = 64) and Castile and León (*n* = 60). The full geographical distribution of participants can be found in Supporting Information S1: Appendix S1.

### 2.2 Data Collection

Participation consisted of completing the online D1ANAS questionnaire, an ad hoc survey developed for this project. The questionnaire was completed using the SphinxOnline platform, whose servers are hosted at the coordinating centre of the principal investigator (University of León). Data were collected between 20 May 2025 and 20 July 2025.

The D1ANAS questionnaire is a self‐administered online survey created based on a combination of validated instruments and ad hoc questions for the collection of variables of interest, including sociodemographic, lifestyle‐related, clinical and diabetes‐related variables, comprising a total of 72 assessable items. Prior to accessing the questionnaire, participants were required to read and electronically accept an informed consent form, which included detailed information regarding data processing, confidentiality, and anonymisation procedures.

### 2.3 Disordered Eating Behaviours Assessment

The primary outcome of the study was the risk of DEBs, assessed using the Spanish validated short version of the Eating Problems Survey for Diabetes (DEPS‐R) [20]. The DEPS‐R consists of 16 items scored on a Likert scale, with total scores ranging from 0 to 80. A score ≥ 20 was considered indicative of a positive screening for DEBs.

The DEPS‐R has demonstrated good psychometric properties for the screening of DEBs in patients with T1D [13, 17, 21], and it has been validated in Spanish [20]. Despite the fact that several authors have identified different factor model structures or subscales (i.e., the three factor model [13], consisting of: ‘eating habits’, ‘thinness’ and ‘high‐blood glucose’ or the four factor model, adding ‘restriction’, or even five‐factor), the one‐factor model has currently shown the strongest evidence. Thus, clinicians and researchers will not need to consider individualised treatment based on various scores across different subscales but they will instead be able to use a single overall DEPS‐R score 20 as suggested by DEPS‐R developers [12] for simple and effective screening for disordered eating in patients with T1D [22]. However, some key variables for which there is strong evidence of usefulness in the literature were extracted from the questionnaire in order to obtain a broader view of the results obtained and to facilitate intercomparability with other studies. These variables were: insulin restriction (calculated from questions 4 and 13 of the questionnaire) and the variable of maintaining high blood glucose to lose weight (question 9).

### 2.4 Data Analysis

Descriptive analyses were performed to characterise the study population. Continuous variables were summarised using means and standard deviations (SD) or medians and interquartile ranges (IQR), as appropriate, while categorical variables were described using frequencies (*n*) and percentages (%). Estimates of the prevalence of DEBs were calculated for the total study population and stratified by sex.

Bivariate analyses were conducted to explore differences by sex in different sociodemographic, clinical, and DEPS‐R related variables. Chi‐square tests were used for categorical variables, and after verifying that the sample did not fit normal distribution the U the Mann–Whitney test for comparison of medians was applied for continuous variables.

Linear regression was performed to examine associations between DEPS‐R scores and selected sociodemographic, clinical, and lifestyle variables. Regression coefficients (*β*), 95% confidence intervals (95% CI) and *p*‐values were reported to indicate the direction, magnitude, and significance of associations. Model fit was evaluated using *R* ^2^ and adjusted *R* ^2^. Covariates for the regressions were chosen based on prior literature (theory‐driven approach) and entered simultaneously into a fully adjusted model and were operationalised as follows.

Age was treated as a continuous variable (years), and sex was coded as a binary variable (male/female). Region of residence was collected according to Spanish autonomous communities and used descriptively. Clinical variables included age at diagnosis of T1D and diabetes duration, both expressed in years and treated as continuous variables. Insulin treatment modality was categorised as multiple daily injections (MDI) or continuous subcutaneous insulin infusion (CSII), with CSII considered as the reference category in the model. Daily insulin dose was collected in units per day; however, due to missing values, this variable was not included in the final multivariable model.

Glycaemic control was assessed using the most recent HbA1c value reported by participants, expressed as a percentage (%) and included as a continuous variable. BMI was calculated from self‐reported weight (kg) and height (cm) using the standard formula (kg/m^2^). For analytical purposes, BMI was standardised (zBMI) to allow comparison of effect sizes across variables and was entered as a continuous variable in regression analyses.

Lifestyle‐related variables included perceived health status and sleep perception, both assessed using self‐rated items adapted from the Spanish National Health Survey and categorised as good, fair, or poor. For regression analyses, good perceived health and good sleep perception were used as reference categories. Physical activity level was assessed using the short form of the International Physical Activity Questionnaire (IPAQ‐SF) and categorised as low or moderate/vigorous, with moderate/vigorous activity used as the reference category. Dietary quality was assessed using an ad hoc questionnaire based on the Spanish National Health Survey, and participants were classified into three categories: poor diet, diet needing changes, or healthy diet, according to the Healthy Eating Index (HEI–2015) [23] and the recommendations of the Spanish Society of Community Nutrition [24]. For this purpose, a maximum score of 10 points and a minimum score of 0 according to the frequency of consumption were awarded to each category, so the maximum total score was 100. In this study, on the basis of the total score, two categories were defined: ≤ 80 the diet needs improvements/poor diet and > 80 good diet/healthy diet. A healthy diet was used as the reference category in regression analyses.

All statistical analyses were performed using STATA software (version 19.0; StataCorp LLC, College Station, TX, USA [25]). A two‐sided *p*‐value < 0.05 was considered statistically significant.

### 2.5 Ethical Aspects

The study was conducted in accordance with the Declaration of Helsinki and received approval from the Ethics Committee of the University of León, which acted as the coordinating ethics committee (approval number: ETICA‐ULE‐043‐2025).

All participants provided the same written informed consent before participation. In accordance with Spanish data protection legislation (Law 3/2018), participants aged 14 or over may give their own consent to take part in the survey and to the processing of the data collected.

Data were collected anonymously through an online platform (Sphinx Online) compliant with the European General Data Protection Regulation (Regulation EU 2016/679) and Spanish data protection law (Law 3/2018). The database was securely hosted by the Information and Communications Service of the University of León, ensuring data confidentiality and restricted access.

## 3 Results

### 3.1 Sociodemographic and Clinical Data

Table 1 shows the characteristics of the 451 individuals included. The majority of participants were women (82.0%). The mean age of the study population was 33.9 years (SD 12.2), with the maximum age represented 71 years in men and 68 years in women. The most representative categories of age were 26–39 years (39.9%) and 40–59 years (27.9%) with a mean duration of diabetes of 17.6 years (SD 11.4). The mean HbA1c level was 6.8% (SD 1.2) and regarding insulin treatment, men were more frequently treated with MDI than women (67.9% vs. 44.9%, respectively).

**TABLE 1:** Clinical and sociodemographic characteristics of the sample, by sex.

	Total	Men	Women	*p*‐value^b^
Number of participants (%)	451 (100)	81 (18.0)	370 (82.0)	—
Age (years)	33.9 (12.2)	35.6 (12.8)	33.5 (12.0)	0.087^a^
Age (categories, years, *n*, %)				
< 18	25 (5.5)	8 (9.8)	17 (4.6)	
18–25	107 (23.7)	15 (18.5)	92 (24.9)	
26–39	180 (39.9)	26 (32.1)	154 (41.6)	0.030
40–59	126 (27.9)	27 (33.3)	99 (26.8)	
> 60	13 (2.88)	5 (6.2)	8 (2.2)	
BMI, mean (SD)	24.5 (4.3)	24.4 (4.1)	24.5 (4.4)	0.900^a^
Diabetes duration (years)	17.6 (11.4)	18.9 (12.0)	17.3 (11.3)	0.173^a^
HbA1c (%)	6.8 (1.2)	6.6 (1.1)	6.9 (1.2)	0.085^a^
Insulin units/day^a^	37.1 (18.3)	39.6 (18.9)	36.6 (18.1)	0.109^a^
Treatment				
MDI, *n* (%)	221 (49.0)	55 (67.9)	166 (44.9)	< 0.001
CSII, *n* (%)	230 (51.0)	26 (32.1)	204 (55.1)	

*Note:* Values are presented as mean (standard deviation) or number (percentage).

Abbreviations: BMI, body mass index; CSII, continuous subcutaneous insulin infusion; *M*, mean; MDI, multiple daily injections; SD, standard deviation.

^a^ 7.1% of participants had missing values for this variable.

^b^ *p*‐value showed for assessing of the differences by sex, employing Mann–Whitney *U* test (continuous variables) or chi‐square test (categorical variables).

### 3.2 Disordered Eating Behaviours

The mean total DEPS‐R score in the overall study population was 16.8 (SD 12.1) and the median score was 14 (IQR: 8–22). Table 2 also shows that 31.7% of participants (*n* = 143) scored positively on the DEB screening (≥ 20 points). Marked sex differences were observed, with a significantly higher prevalence of positive DEPS‐R among women (35.4% vs. 14.8%, *p* < 0.001).

**TABLE 2:** DEPS‐R descriptive data were obtained for the whole sample and stratified by sex.

	Total (*N* = 451)	Males (*n* = 81)	Females (*n* = 370)	*p*‐value^b^
	Median (IQR)/*N* (%)	Median (IQR)/*N* (%)	Median (IQR)/*N* (%)	
DEPS‐R				
Total score	14 (8–22)	10 (6–15)	15 (8–24)	< 0.001
Score ≥ 20, *n* (%)	143 (31.7)	12 (14.8)	131 (35.4)	< 0.001
Insulin restriction, *n* (%)	165 (36.6)	33 (40.7)	132 (35.7)	0.391
DEPS‐R score excluding insulin‐restriction^a^	13 (7–21)	9 (6–13)	14 (8–22)	< 0.001

*Note:* A DEPS‐R score ≥ 20 was used to define a positive screening for disordered eating behaviour (DEB). The variable insulin restriction was calculated using the items 4 (‘When I over‐eat, I do not take enough insulin to cover the food’) and 13 (‘After I overeat, I skip my next insulin dose’) of the Spanish version of DEPS‐R, a score indicating reducing or skipping at least ‘sometimes’ in both questions was considered as intentional insulin restriction.

^a^ For this variable, items 4 and 13 were eliminated for the global score.

^b^ The *p*‐value shown is the result of the *U* de–Whitney test for continuous variables and the Chi‐squared test for categorical variables by sex.

Insulin restriction behaviours were reported by 36.6% of participants and were similarly prevalent in both sexes (40.7% vs. 35.7%, respectively; *p* = 0.391).

When insulin‐related items were excluded from the DEPS‐R total score, the median score in the overall sample remained largely unchanged (13 points [IQR: 7–21]), but sex differences persisted.

### 3.3 Associated Factors With Disordered Eating Behaviours

**FIGURE 2:**
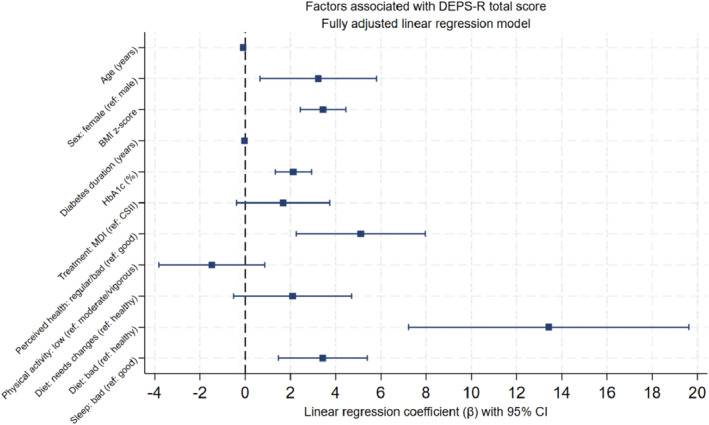
Factors associated with the DEPS‐R total score. Forest plot showing regression coefficients (*β*) and 95% confidence intervals from the fully adjusted linear regression model examining sociodemographic, clinical, and lifestyle‐related factors associated with DEPS‐R total score in the whole sample (*N* = 451). Positive *β* values indicate higher DEPS‐R scores, whereas negative values indicate lower scores than the reference category. The vertical dashed line represents the null value (*β* = 0). BMI, body mass index, CSII, continuous subcutaneous insulin infusion; MDI, multiple daily injections.

The results of linear regression analysis to examine the associations between the DEPS‐R total score and sociodemographic, clinical, and lifestyle‐related variables in the whole sample are shown in Figure 2.

Female sex was significantly associated with higher DEPS‐R scores compared with male sex (*β* = 3.23, 95% CI = 0.65−5.82, *p* = 0.014), as well as higher zBMI (*β* = 3.44, 95% CI = 2.44−4.44, *p* < 0.001). Higher HbA1c percentage was positively associated with higher DEPS‐R scores (*β* = 2.12, 95% CI = 1.32−2.93, *p* < 0.001). The MDI versus CSII insulin treatment modality showed a trend towards a higher DEPS‐R score risk, although this difference was not statistically significant. Lifestyle‐related factors also showed strong associations with higher DEPS‐R scores. Compared to participants who reported good perceived health, those who reported fair or poor perceived health had significantly higher DEPS‐R scores (*β* = 5.11, 95% CI = 2.25−7.96, *p* < 0.001). Regarding dietary quality, reporting a poor diet was associated with significantly higher DEPS‐R scores compared with reporting a healthy diet (*β* = 13.42, 95% CI = 7.23−19.61, *p* < 0.001).

Similarly, poor sleep perception was associated with higher DEPS‐R scores compared with good perception (*β* = 3.43, 95% CI = 1.46−5.39, *p* = 0.001).

Overall, the fully adjusted model explained 29.6% of the variance in DEPS‐R total scores (*R* ^2^ = 0.296; adjusted *R* ^2^ = 0.278).

## 4 Discussion

In this nationwide cross‐sectional study of a sample of people with type 1 diabetes in Spain, approximately one‐third of participants screened positive for disordered eating behaviours using the DEPS‐R. This estimate is a bit higher compared to those reported in previous studies [3, 4, 7]. Higher DEPS‐R scores were independently associated with poorer glycaemic control, higher zBMI and several lifestyle‐related factors, including poorer perceived health status, poorer sleep perception and lower diet quality [26, 27, 28].

A growing body of literature indicates that DEBs are substantially more prevalent in people with T1D than in the general population [1, 3, 4]. Population‐based longitudinal data further underscore the clinical burden of DEBs in T1D. For example, in a nationwide Finnish cohort, the incidence of clinically diagnosed eating disorders was more than twofold higher in individuals with T1D compared with matched controls; however, access to specialist care for eating disorders was lower, highlighting a clinically relevant therapeutic gap [16].

Consistent with prior literature [7, 11, 13], we observed a significantly higher prevalence of DEBs among women compared with men (35.4% vs. 14.8%). Despite this, the high prevalence of DEBs among males in this population highlights that DEBs are not restricted to females in this population and may be under‐recognised in male patients [2, 11, 13, 29].

We found that omitting or reducing insulin was a common behaviour in both men and women (reported by 36.6% of participants), but it represents only one aspect of DEBs in diabetes, and while some people use it to control weight or caloric intake, not all individuals at risk for eating disorders are likely to engage in this behaviour. In other studies, eating disorders and insulin underdosing have been reported as frequent and notably, not necessarily, showing a marked gender difference [30]. Importantly, DEPS‐R scores excluding insulin‐related items remained significantly higher in women, indicating that the overall burden of DEBs in this sample is largely driven by classic eating‐related psychopathology. These findings align with previous studies highlighting the coexistence of weight concern, loss of control overeating and maladaptive eating attitudes in people with T1D [6, 31].

Regarding the treatment method, individuals using MDI had higher DEPS‐R scores than those using CSII (non‐significant association). This finding is consistent with previous studies reporting no clear differences in DEBs between MDI and CSII users in people with T1D [32, 33, 34].

Regarding lifestyle habits, poor diet quality was strongly associated with higher DEPS‐R scores, suggesting that a more flexible and adaptive approach to eating may be linked to lower risk of DEBs. In line with this interpretation, interventions targeting diet quality in youth with T1D did not increase DEBs, while higher DEPS‐R scores were independently associated with higher HbA1c and greater time in hyperglycemia [35]. Likewise, a recent scoping review suggests that carbohydrate counting is not intrinsically associated with increased DEBs risk and may even be protective in some contexts [36].

Poor sleep perception and poorer perceived health status were both associated with higher DEPS‐R scores. Sleep disturbances in T1D may reflect nocturnal glycaemic instability, having been associated with higher HbA1c [37] and worse glycaemic control, and with a high prevalence of obstructive sleep apnoea [28, 38]. This aligns with evidence linking psychological distress, impaired well‐being, and sleep disturbances to maladaptive eating behaviours in people with T1D [2, 28, 37].

The practice of less physical activity was associated with a lower score on the DEPS‐R, although no statistical significance was found. This could be explained by the fact that physical activity is part of both healthy self‐management [39] and unhealthy compensatory behaviours, particularly in eating disorders in the general population [40] where adaptive and maladaptive exercise often co‐exist. However, further investigation is needed in this area in people with T1D.

### 4.1 Implications for Clinical Practice

These findings have important implications for clinical nutrition practice. A recent systematic review about the effectiveness of programs to prevent EDs in people with T1D highlighted the lack of studies in this area, particularly well‐powered randomised controlled [41]. Exposure to DEBs is common in adults with T1D (one third of participants) and clinically relevant even in individuals with apparently acceptable glycaemic control.

In this perspective, screening for DEBs should be routinely incorporated into adult T1D care, using validated diabetes‐specific tools such as DEPS‐R. Importantly, DEBs were observed even among individuals with apparently acceptable glycaemic control, suggesting that these behaviours may remain under‐recognised in routine diabetes care. Our findings also underscore the importance of considering lifestyle and psychosocial factors as part of comprehensive diabetes management. From a clinical nutrition perspective, a multidisciplinary approach addressing nutritional behaviours, metabolic control, sleep quality, and psychosocial well‐being is essential for early identification and intervention. This is reinforced by current Standards of Care, which explicitly recommend regular psychosocial screening, including DEBs, especially in the presence of unexplained hypoglycemia, recurrent ketoacidosis or marked glycaemic variability [42]. Early detection and integrated management may therefore represent key strategies to improve both metabolic control and quality of life in adults with T1D.

### 4.2 Limitations and Strengths

Despite the significant results found in this work, it has limitations including the cross‐sectional design reliance on self‐reported data and the use of a screening instrument rather than formal psychiatric diagnoses, which prevent causal inference. A large quantity of women participated in our study, however, we hope to control this limitation by the stratification by sex in the analysis. Another limitation is the unequal age distribution within the sample. Specifically, people older than 60 years were a low percentage of the total sample (2.9%). This fact may be due to the use of new technologies to conduct the study, as part of this population has more limited access to social media, which could have led to this age group being under‐represented. However, this limitation has been taken into account and age has been included in the analysis.

The sample selection has also limitations, as this is a convenience sample mainly through social media, patient associations and a snowball approach. Notwithstanding, T1D people are a minority population that is difficult to access, so it can be concluded that our sample is valid for the hypothesis proposed.

Despite these limitations, this study also has several strengths. To our knowledge, this is the first nationwide study assessing DEBs in adults with T1D in Spain using a validated diabetes‐specific screening instrument. The relatively large sample size and the inclusion of participants from different regions of the country enhance the relevance of the findings in a real‐world context. In addition, the study examined a broad range of clinical, lifestyle, and psychosocial variables, allowing a more comprehensive understanding of factors associated with disordered eating behaviours in this population. Another strength is the incorporation of patient and public involvement (PPI) principles in the development of the D1ANAS project, which may improve the relevance and applicability of the research to the needs of people living with T1D.

## 5 Conclusion

Disordered eating behaviours affect approximately one third of people with T1D in this Spanish sample and are associated with a complex interplay of metabolic, lifestyle and psychosocial factors. Higher risks were independently associated with poorer glycaemic control, higher body mass index and lifestyle factors, including poorer diet quality, poorer perceived health status and poorer sleep perception. Although women showed a higher prevalence of DEBs, a substantial proportion of men also screened positive, highlighting that these behaviours may be under‐recognised in male patients. Together, these findings highlight the need for routine screening for DEBs in adult T1D care and support a multidisciplinary approach that integrates nutritional, metabolic, behavioural and psychosocial aspects to improve both diabetes management and patients' well‐being.

Future longitudinal studies are warranted to clarify the temporal relationships between disordered eating behaviours, lifestyle factors, and metabolic outcomes in adults with type 1 diabetes.

## Author Contributions

**Lorena Botella‐Juan:** conceptualization, methodology, formal analysis, investigation, data curation, writing – original draft, supervision. **Inmaculada Aguilera‐Buenosvinos:** methodology, formal analysis, writing – original draft, writing – review and editing. **María Antequera‐González:** conceptualization, investigation, writing – original draft, writing – review and editing. **Laura Álvarez‐Álvarez:** methodology, visualization, writing – original draft, writing – review and editing. **María Rubín‐García:** formal analysis, methodology, writing – original draft, writing – review and editing. **María Romeo‐Santos:** investigation, visualization, writing – review and editing. **Ana Segura‐Grau:** supervision, visualization, writing – review and editing. **María D. Ballesteros‐Pomar:** conceptualization, investigation, writing – review and editing. **Antonio José Molina:** resources, supervision, writing – review and editing. **Vicente Martín‐Sánchez:** resources, supervision, writing – review and editing.

## Funding

The authors have nothing to report.

## Ethics Statement

The study was conducted in accordance with the Declaration of Helsinki and received approval from the Ethics Committee of the University of León, which acted as the coordinating ethics committee (approval number: ETICA‐ULE‐043‐2025).

## Consent

All participants provided written informed consent prior to participation.

## Conflicts of Interest

The authors declare no conflicts of interest.

## Authorship Statement

All individuals who meet the authorship criteria have been included as authors, and non‐eligible authors have been excluded from the author list.

## Supporting information


Supporting Information S1

## Data Availability

D1ANAS data are available upon a justified petition to the corresponding author request.
