# Supplementary material for: Disordered Eating Behaviours and Associated Factors Among People With Type 1 Diabetes: A Cross‐Sectional Study in Spain—D1ANAS Project
Source: Diabetes Metab Res Rev. 2026 Jul 15;42(5):e70204. doi: 10.1002/dmrr.70204 (PMC13372235; doi:10.1002/dmrr.70204)
Supplement: Supplementary file 1 — Supporting Information S1 [file DMRR-42-e70204-s001.docx]

**SUPPLEMENTARY APPENDIX 1**

**
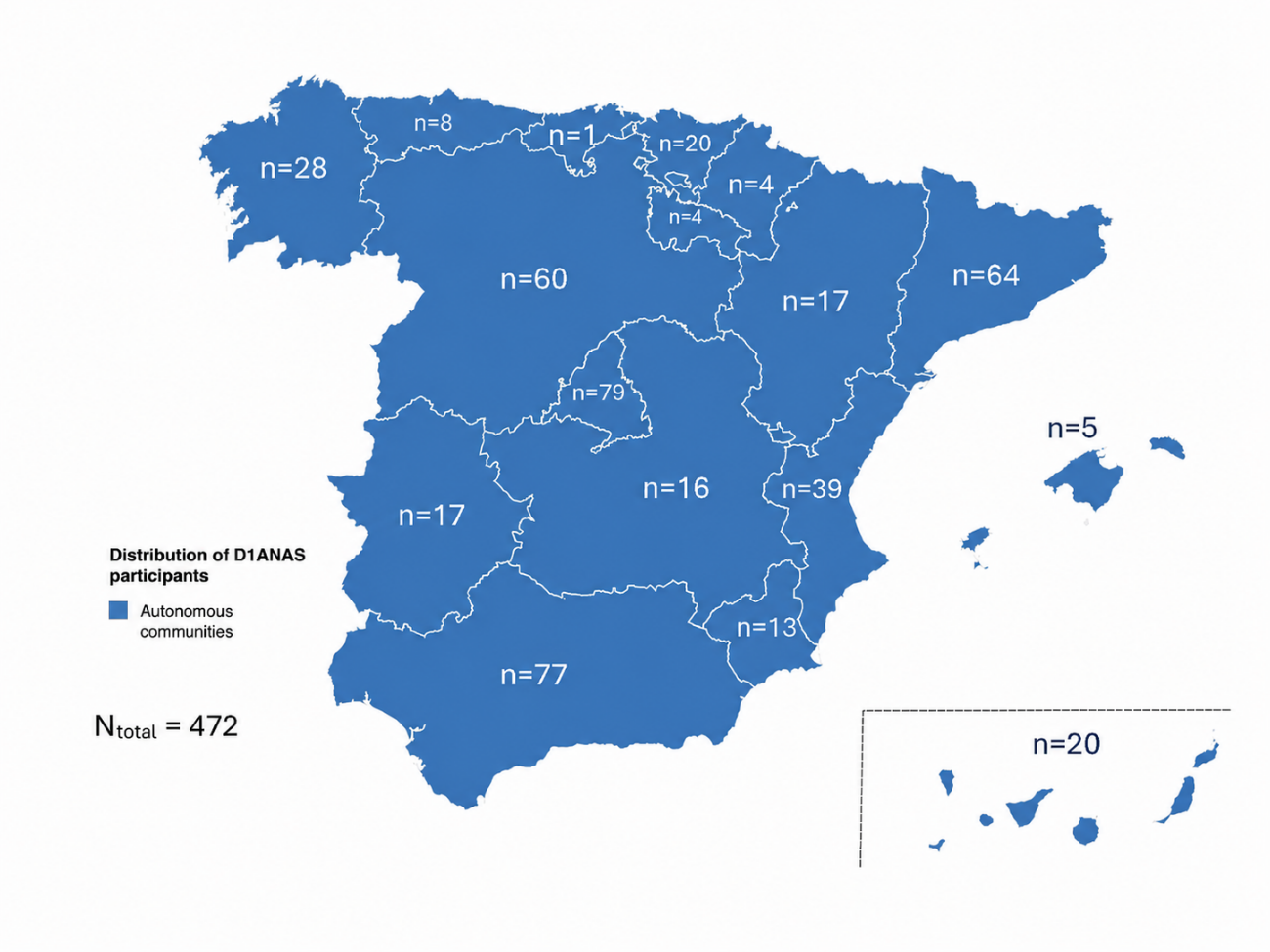
**

**Supplementary appendix 1.** Distribution of the participants of the D1ANAS Project, showing the number (n) of participants from each autonomous community/region in the Spain map.
